# Improvement of Electrical Conductivity of In Situ Iodine-Doped Polypyrrole Film Using Atmospheric Pressure Plasma Reactor with Capillary Electrodes

**DOI:** 10.3390/nano14050468

**Published:** 2024-03-04

**Authors:** Eun Young Jung, Salman Khalil, Hyojun Jang, Habeeb Olaitan Suleiman, Jae Young Kim, Bhum Jae Shin, Heung-Sik Tae, Choon-Sang Park

**Affiliations:** 1 The Institute of Electronic Technology, College of IT Engineering, Kyungpook National University, Daegu 41566, Republic of Korea; eyjung@knu.ac.kr; 2 School of Electronic and Electrical Engineering, College of IT Engineering, Kyungpook National University, Daegu 41566, Republic of Korea; salmank.durrani@knu.ac.kr (S.K.); bs00201@knu.ac.kr (H.J.); suleiman.habeeb16@knu.ac.kr (H.O.S.); jyk@knu.ac.kr (J.Y.K.); 3 Department of Electronics Engineering, Sejong University, Seoul 05006, Republic of Korea; hahusbj@sejong.ac.kr; 4 Electrical Engineering, Milligan University, Johnson City, TN 37682, USA

**Keywords:** atmospheric pressure plasma, capillary electrodes, in situ heated iodine doping, polypyrrole nanostructure film

## Abstract

To improve the electrical conductivity of polypyrrole (PPy) nanostructure film through in situ iodine (I_2_) doping, this study proposes an atmospheric pressure plasma reactor (APPR) where heated I_2_ dopant vapor is fed through capillary electrodes that serve as electrodes for discharge ignition. A large amount of the heated I_2_ vapor introduced into the reactor separately from a monomer gas can be effectively activated by an intense plasma via capillary electrodes. In particular, intensive plasma is obtained by properly adjusting the bluff body position in the APPR. Based on the ICCD and OES results, the I_2_ vapor injected through the capillary nozzle electrode is observed to form I_2_ charge species. The formed I_2_ species could directly participate in growing in situ I_2_-doped PPy films. Thus, in situ I_2_-doped PPy nanostructure films grown using the proposed APPR exhibit higher thicknesses of 15.3 μm and good electrical conductivities, compared to the corresponding non-doped films.

## 1. Introduction

Conductive polymers (CPs), such as polypyrrole (PPy), polythiophene, polyacene, and polyaniline, generally exhibit the structural characteristics of π-conjugated polymers. These conjugated polymers possess electrical conductivity properties when doped to form charge carriers [1,2,3,4]. Thus, CPs are widely studied and have attracted great interest for their applicability to various devices in the optoelectronic industry, such as polymer light-emitting diodes, solar cells, energy storage devices, and sensors [1,2,3,4].

To synthesize CPs with excellent electrical properties using conventional plasma processes, doping is performed using materials such as iodine (I_2_), chlorine, hydrogen chloride, and iron trichloride [5,6,7,8,9,10,11,12,13,14]. Many studies have directly injected I_2_ into the reaction chamber during plasma polymerization using in situ techniques for both low-pressure and atmospheric-pressure (AP) plasmas [14,15,16,17,18,19,20,21,22,23,24]. To achieve high concentration and sufficient I_2_ doping, the amount of I_2_ vapor flow must be considerably increased during the polymer film growth [25]. Conventional in situ I_2_ doping typically uses solid I_2_ particles to inject I_2_ vapor under room-temperature conditions [12,20,21,22,23,24]. Solid I_2_ particles have a high vapor pressure and a low sublimation temperature [26], making it difficult to considerably increase the I_2_ vapor flow for achieving high doping under room-temperature conditions. Employing heating facilitates I_2_ vaporization, enabling high doping. Implementing heating in in situ I_2_ doping allows the injection of a large amount of heated I_2_ vapor into the plasma reaction chamber during the polymer film growth. In this case, however, achieving an efficient recombination reaction necessitates the simultaneous production of an intense plasma, which can sufficiently activate a large amount of the heated I_2_ vapor introduced into the plasma reaction chamber, as well as the minimization of the excessive monomer vapor cracking caused by this intense plasma. The contamination induced by injecting a large amount of the heated I_2_ vapor as well as the monomer vapor flow into the reaction chamber must also be minimized [22,27]. Satisfying these conditions requires the consideration of an effective powered electrode design, including gas feeding tubes with an effective design in the atmospheric-pressure plasma (APP) systems. 

However, a few studies have investigated the injection of in situ heated I_2_ vapor during polymer film deposition for both low-pressure and AP plasmas. Moreover, because the monomer and heated I_2_ vapors are injected through the same gas-feeding tube, contamination occurs inside the gas feeding tube of conventional APP devices [14,15,16], which use a micro-sized capillary tube for gas feeding. Nonetheless, APP systems are simple structures used in ambient air. Hence, the heating temperature can easily be increased to produce a large amount of heated I_2_ vapor. We previously proposed an APP device with a guide-tube and bluff-body system [14,15,16]. However, overcoming the problem of contamination requires a new type of APP device with a separate gas-feeding tube. A new powered-electrode design is necessary for activating a large amount of the I_2_ vapor to achieve an efficient recombination reaction with the monomer vapor. 

To improve the conductivity of PPy films through in situ I_2_ doping, this study proposes an atmospheric pressure plasma reactor (APPR) with capillary electrodes where the heated I_2_ dopant vapor is fed into the reactor via capillary electrodes that serve as electrodes for igniting discharges. In the proposed doping, a large amount of the heated I_2_ vapor is introduced separately from the monomer gas through capillary electrodes into the APPR. Unlike conventional powered electrode configurations, the capillary electrodes for feeding the I_2_ vapor are used as powered electrodes. Furthermore, the bluff body position is optimized with respect to the glass guide tube of the APPR for the growth of in situ I_2_-doped PPy nanostructure films by producing an intense plasma near the region where a large amount of the heated I_2_ vapor is injected. Using a digital camera, optical emission spectroscopy (OES), and an intensified charge-coupled device (ICCD), the plasma is investigated, considering three bluff body positions inside the glass guide tube. The characteristics of the PPy films grown in the proposed APPR and two in situ doping conditions (i.e., no doping (Case I) and in situ I_2_ doping at a hot plate temperature of 35 °C (Case II)) are examined using Fourier-transform infrared spectroscopy (FTIR), scanning electron microscopy with energy-dispersive X-ray spectroscopy (SEM-EDX), and field-emission scanning electron microscopy (FE-SEM). For Case II, the electrical conductivity of the film grown on a glass substrate in the APPR is measured using a four-point probe.

## 2. Materials and Methods

### 2.1. Experimental Setup 

**Figure 1:**
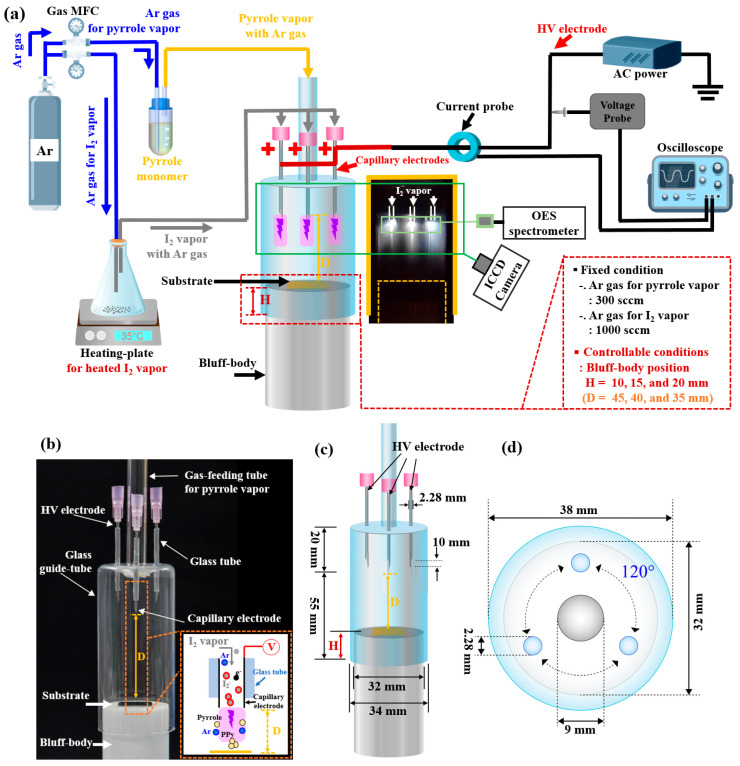
(**a**) Experimental setup of an APPR with capillary electrodes for in situ I_2_ doping, (**b**) photo image, (**c**) schematic diagram, and (**d**) top plane view of the APPR.

Figure 1 presents the experimental setup of an APPR with capillary electrodes for growing in situ I_2_-doped PPy nanostructure film. For both cases, the APPR comprised a glass tube for gas feeding, a glass guide tube, and a bluff body for generating plasma. The capillary electrodes comprised three nozzle electrodes with stainless steel syringe nozzles (Figure 1a–c). An alternating-current (AC) high voltage was commonly applied to the equipotential capillary electrodes. 

As the APPR device used capillary electrodes, a large amount of the heated I_2_ vapor was separately introduced from a monomer gas flow for the PPy film growth. The three capillary electrodes were covered with a glass tube of 2.28 mm diameter. The electrode tips protruded 10 mm from the end of the glass tube. The APPR device used the capillary electrodes, that is, the three nozzle electrodes, as pin-type electrodes without a ground electrode. These electrodes were exposed within the plasma discharge region inside the plasma reactor of the glass guide tube. Thus, because the electrodes were exposed to the discharge region, they can be easily cleaned, and the heating temperature can be easily changed, even under contamination (Figure 1b,c). The three capillary electrodes were arranged at 120° angles with each other in the horizontal plane on top of the glass guide tube (Figure 1d). 

The I_2_ vapor for doping was heated on a hotplate using a glass flask containing 10 g of solid I_2_ pellets at a slightly low temperature below 50 °C and 100 °C, in which the solid-gas phase was maintained to prevent the formation of a condensed phase [28,29,30]. 

Notably, the heated I_2_ vapor was set to a hot plate temperature of 35 °C, such that the PPy polymer structure could be maintained. At an in situ heating temperature of over 50 °C, the deposited PPy film surfaces smoothly changed because the PPy nanoparticles (NPs) on the film surfaces melted and disappeared owing to the heated I_2_ vapor formed, which was transported using argon (Ar) gas at a 1000 sccm flow rate during the PPy film growth. Herein, the bluff body position with respect to the glass guide tube was an important factor influencing the formation of an intense plasma for the PPy film growth. The bluff body was fabricated using polytetrafluoroethylene, intending to hold a substrate for the polymer film growth. This bluff body is electrically floating and is in a virtual ground state. A 40 mL pyrrole (molecular weight = 67.09 g·mol^−1^) liquid monomer was vaporized using a glass bubbler by passing Ar gas at a flow rate of 300 sccm for pyrrole vapor. Subsequently, the obtained vapor gas was flowed through a glass gas-feeding tube in the APPR for PPy film growth. The PPy film was coated on glass and silicon substrates using the APPR. The discharge gas was provided at a 1000 sccm gas flow rate. High-purity Ar gas (99.999%) was used to form the intense plasma under ambient air in the closed guide tube of APPR for in situ I_2_-doped PPy nanostructure film growth. Thus, the air, including N_2_, would be employed inside the plasma reactor during the PPy film growth, and then the discharge would be produced in the presence of air gases. Some of the N_2_ from the air would be transformed into excited N_2_ species by plasma energy. A bipolar sinusoidal waveform was applied to the powered electrodes. Table 1 presents the experimental conditions used for synthesizing in situ I_2_-doped PPy films using the proposed APPR.

### 2.2. Discharge Voltage and Current Pulse Signal Analysis 

An inverter-type power supply was used to operate the APPR system owing to its high voltage and low current requirements. The electrical characteristics during the plasma discharge were identified by monitoring the voltage and current waveforms displayed on a digital oscilloscope using high-power voltage and current probes. A high-voltage probe (P6015A, Tektronix Inc., Beaverton, OR, USA) was used to monitor the applied voltage waveform. A commonly known instrument current transformer (Model 4100, Pearson Electronics Inc., Palo Alto, CA, USA) (in the form of a coil) was used as the current probe. Because this current transformer operates in a series connection as an instrument transformer, it was used by passing a high-voltage cable through a central hole.

### 2.3. ICCD and Digital Camera

The intensified charge-coupled device (ICCD) images and photos of the plasma produced in the APPR were obtained using an ICCD and a digital camera, respectively. 

To evaluate the horizontal and vertical distributions of the formed plasma in the APPR for PPy film growth, the produced plasma discharge was evaluated using an ICCD camera (PI–MAX 2, Princeton Instruments, Trenton, NJ, USA) in both shutter modes with a 1 µs exposure time.

### 2.4. OES

The behaviors of the excited species (e.g., nitrogen (N_2_) and Ar species) in the formed plasma discharge were analyzed through optical emission spectroscopy (OES) implemented using an optical spectrometer (USB–2000+, Ocean Optics, Orlando, FL, USA) with an optical resolution of 0.1~10.0 nm. OES spectra were obtained with a wavelength interval of 0.22 nm in the wavelength range from 200 to 900 nm. To obtain the plasma emission from the bulk plasma within the glass guide tube, the detecting probe focused on the plasma emission region at 3 mm apart from the tip of the capillary electrodes. The detecting probe was placed 5 mm away from the outside of the glass guide tube. Under this measurement position, a 100 µm optical fiber and an integration time of 1 ms were determined to obtain the OES spectra with a high S/N ratio from the bulk plasma.

### 2.5. FE-SEM and EDX

The plane-view and cross-sectional images of the PPy films were examined on silicon substrates through field-emission scanning electron microscopy (FE-SEM; SU8220, Hitachi High-Technologies, Tokyo, Japan) with an acceleration voltage and an electron emission current of 3 kV and 10 μA, respectively. The PPy films were covered with conductive platinum before being loaded into the reaction chamber. The growth rate of the PPy films was calculated by measuring their thickness based on the cross-section image obtained through FE-SEM. The component distribution in the cross-section of the in situ I_2_-doped PPy nanostructure films grown on a silicon substrate was analyzed using an energy-dispersive X-ray spectroscopy (EDX, Horiba, Kyoto, Japan) detector attached to the FE-SEM instrument.

### 2.6. FTIR Spectroscopy

The prepared samples were analyzed using Fourier-transform infrared spectroscopy (FTIR; Vertex 70, Bruker, Germany) at the Korea Basic Science Institute (Daegu, Republic of Korea) to characterize the crystalline phases of the in situ I_2_-doped PPy nanostructure films. The FTIR spectra were measured in the attenuated total reflection mode by averaging 128 scans for a wavenumber range of 670–4000 cm^−1^ and a spectral resolution of 0.6 cm^−1^.

### 2.7. Four-Point Probe

The electrical sheet resistance (R_s_) of the in situ I_2_-doped PPy nanostructure films was measured at room temperature under ambient air conditions with a humidity of approximately 40% using a four-point probe (T2001A3, Ossila, Sheffield, UK). The average value of electrical R_s_ for each sample was obtained by averaging five measurements. A 2 × 2 cm^2^ square was cut from the glass substrate. The electrical R_s_ was measured by applying a direct current through the two outer probes. Upon contacting the four-point probe to the PPy film surface, a fixed current of 10 µA was then supplied to the two outer probes, and the voltage difference between the two inner probes was measured. The electrical R_s_ was obtained when measuring the voltage drop [22]. If the film thickness is known, then the obtained electrical R_s_ can be used to calculate the film electrical conductivity based on Equations (1) and (2).

(1) $$R_{s}=\rho \;\frac{L}{R_{s}\;A}=\frac{1}{\sigma }\times \frac{L}{R_{s}\;A}$$

(2) $$\sigma =\;\frac{L}{R_{s}\;A}=\;\frac{L}{R_{s}\;W\;T_{f}}$$

where R_s_ is the electrical sheet resistance, *σ* is the film electrical conductivity, *ρ* is the resistivity, *L* is the sample length, *W* denotes the width, and *T_f_* is the film thickness [31].

## 3. Results

### 3.1. Plasma Characterization 

#### 3.1.1. Photos and ICCD Images of the Produced Plasma Discharge

**Figure 2:**
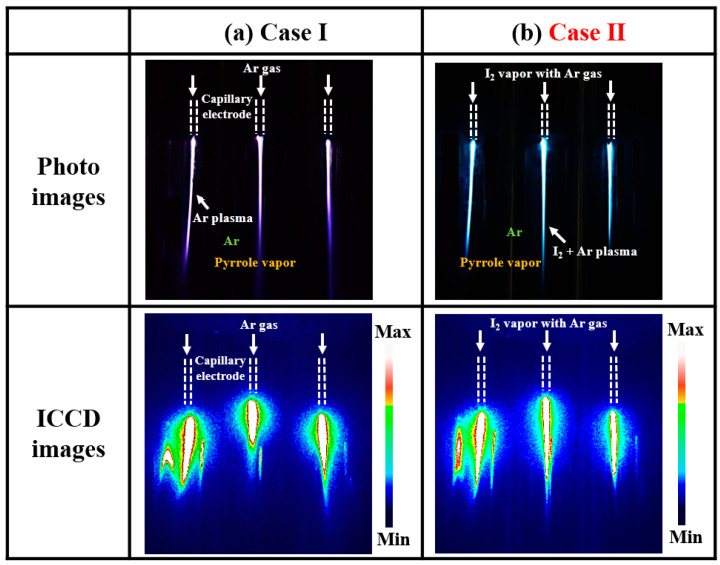
(**a**) Photos and (**b**) ICCD images of the plasma discharge produced in the proposed APPR under the two conditions of no doping (Case I) and in situ I_2_ doping at hot plate temperature of 35 °C (Case II).

Figure 2a,b present the photos and ICCD images of the plasma discharge produced in the APPR with capillary electrodes under two different conditions: no doping (Case I) and in situ heated I_2_ doping at a hot plate temperature of 35 °C (Case II). Both cases showed that the plasma discharge formed well from all the capillary electrodes, even if the capillary electrodes comprising three nozzle electrodes were commonly connected to the applied high-voltage power under a floating bluff body in virtual ground state. 

Figure 2a presents the photos and ICCD images of the plasma generated through a reaction between the Ar gas and pyrrole vapor without I_2_ vapor injection. The generated plasma exhibited purple and pink colors. An intense plasma was formed at the capillary electrode tip owing to the high electric field strength between the electrode tip and substrate. As shown in Figure 2b, an I_2_ plasma was produced through a reaction among the Ar gas, pyrrole vapor, and injected I_2_ vapor. This I_2_ plasma exhibited a bluish-green color. 

From these experimental results, the I_2_ vapor injected through the capillary nozzle electrode is observed to form an I_2_ charge species near the tip of the nozzle electrode. The formed I_2_ species could directly participate in dissociation and ionization reactions during in situ I_2_-doped PPy nanostructure film growth [14,32,33,34,35]. The distance between the substrate and powered electrodes, to which a high voltage is applied, is an important parameter for producing an intense plasma in the APPR for polymer film growth. Our previous experimental results for polyaniline and PPy thin-film depositions revealed a 10 mm bluff body position to be the appropriate condition. Notably, the optimal bluff body position may change, depending on changes in the monomer material and APPR configuration [14]. Therefore, when changing the monomer or vapor conditions, the distance between the plasma source and substrate must be optimized for the polymer film growth by adjusting the bluff body position with respect to the glass guide tube. The distance must be optimized by changing the plasma device structure. Accordingly, three cases of the bluff body position inside the glass guide tube were examined to produce an intense plasma for the in situ I_2_-doped PPy nanostructure film growth using a digital camera and an ICCD.

**Figure 3:**
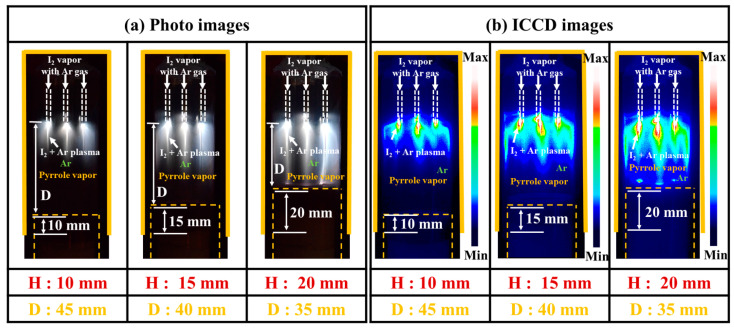
(**a**) Photos and (**b**) ICCD images of the plasma discharge produced in the APPR with three bluff body positions with respect to the glass guide tube.

Figure 3a,b present the photos and ICCD images of the plasma discharge generated in the APPR. When the bluff body positions were changed from 10 to 15 mm, and then to 20 mm with respect to the glass guide tube, the distance (D) between electrode tip and substrate was decreased from 45 to 40 mm, and then to 35 mm. As the D decreased, the produced plasma became intense and exhibited higher intensities due to an active ionization and excitation reaction between short distances. The plasma was strongly ignited at the outside of the capillary electrode tip under a floating bluff body in a virtual ground state, to which a high voltage was applied and spread in a vertical direction inside the glass guide tube [32,33,34,35,36,37,38,39]. When the D decreased (i.e., 45, 40, and 35 mm), the plasma discharge was strongly produced owing to a high static pressure and secondary flow motions (Figure 3a,b) [36]. Moreover, the length of the plasma increased from 10 to 35 mm due to the ionization reaction caused by the high gas pressure in the closed guide tube, decreasing the D [36]. For 45 mm of D, the plasma discharge was weak in a small local area. For 35 mm of D, the produced plasma was intense enough to thermally damage the substrate, melting the surface of the deposited polymer film owing to ion bombardment in the coupling condition (Figure 3a,b) [36]. The experimental results showed that the optimum D for achieving intense plasma was 40 mm under a 1000 sccm Ar flow rate for the heated I_2_ vapor and under a 300 sccm Ar flow rate for the pyrrole vapor.

#### 3.1.2. OES Results with Regard to the Produced Plasma Discharge

**Figure 4:**
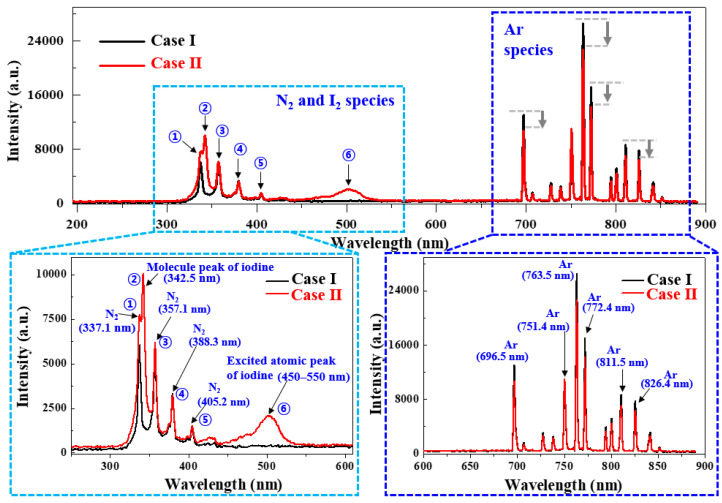
OES spectra of the APPR for the two cases of no doping (Case I) and in situ I_2_ doping at a hot plate temperature of 35 °C (Case II).

OES was employed to compare and examine the excited species (e.g., N_2_ and Ar peaks) formed in the APPR for the two cases as well as analyze the molecular and excited atomic emission of I_2_. Figure 4 shows the OES spectra in the wavelength region of 300 to 900 nm for the produced plasma in the APPR for both cases. Several nitrogen second positive system (N_2_; 337.1, 357.1, 388.3, and 405.2 nm), oxygen (OH: 308 nm and O_3_: 844.1 nm), and Ar peaks (Ar; 696.5, 751.4, 763.5, 772.4, 811.5, and 826.4 nm) were observed in the OES spectra, as shown in Figure 4 [36]. The in situ I_2_-doped PPy film growth was performed under ambient air in the APPR. Accordingly, the air, including N_2_, would be employed in the plasma reactor during the PPy film growth. The N_2_ peaks come from the air plus the pyrrole monomer, and the Ar peaks originate from the plasma produced by using an Ar gas. When the I_2_ vapor was injected, the peaks corresponding to the molecular and excited atomic emission of I_2_ were observed at 347 nm and 450–550 nm (broad peak), respectively [30,40]. 

The OES results showed that when the heated I_2_ vapor was injected during in situ I_2_ doping, the intensity of the N_2_ peak at 337.1 nm increased. For Case II, when injecting the I_2_ vapor for in situ I_2_ doping, since the injected heated I_2_ vapor has a low ionization energy (10 eV), an I_2_ plasma is generated due to a high electric field induced by the nozzle electrode [30]. Under a high electric field condition, the number of high-energy electrons would increase, and, the high-energy electrons would excite the N_2_ species or Ar gas through inelastic collisions. This presence of high-energy electrons in I_2_ plasma causes inelastic collisions between these electrons and N_2_ species, contributing to the increase in the N_2_ peak at 337.1 nm in Figure 4. This N_2_ peak at 337.1 nm corresponds to the emission intensities of the nitrogen band of the second positive system at 337.1 nm [41,42,43,44]. In addition, as the residence time of inflowing N_2_ gas increases in a closed guide tube, N_2_ gas actively collides with Ar gas and electrons. So, the increase in N_2_ peak is in a nitrogen-rich condition in a closed guide tube under the same Ar gas flow. This increase in the N_2_ peak intensity at 337.1 nm was attributable to the inelastic collisions between electrons and N_2_ species. This intensity increment of the N_2_ peak at 337.1 nm indicated the nitrogen-rich condition in the nucleation region, confirming that the monomers can be fully recombined for the in situ I_2_-doped PPy film growth [36]. Notably, the intensity increment of the N_2_ peak at 337.1 nm and the amount of I_2_ species can affect the deposition properties (e.g., polymerization degree for cross-linking, film thickness, and morphology) [45,46]. For Case II, the Ar peak intensity was observed to be weakened, meaning that the Ar plasma has low energy, thereby minimizing the destruction of the pyrrole monomer. Moreover, the PPy film growth was performed under ambient air in the APPR, and therefore, air, including N_2_, would be employed in the APPR during the PPy film growth. Some of the N_2_ from the air would be transformed into excited N_2_ species owing to the plasma energy [44]. When injecting the I_2_ vapor for in situ I_2_ doping, the N_2_ peaks would be increased by high-energy electrons in I_2_ plasma through an inelastic collision between electrons and Ar gas in Figure 4. Notably, an increase in the N_2_ peak at 337.1 nm can contribute to improving the intensity of all major peaks, including those corresponding to the functional groups of the PPy ring structure [44]. The formed I_2_ peaks resulted from I_2_ species, such as molecular and atomic emission of I_2_, which was formed through an ionization reaction by an I_2_ plasma from the heated I_2_ vapor injection [39]. In other words, the injected I_2_ vapor contributed to the formation of the I_2_ plasma by I_2_ species through an ionization reaction near the capillary electrode [14]. These OES results were in good agreement with the photos and ICCD images of the I_2_ plasma presented in Figure 2. Based on the experimental results of Figure 2, Figure 3 and Figure 4, the deposition conditions were optimized for in situ I_2_-doped PPy nanostructure film. 

**Figure 5:**
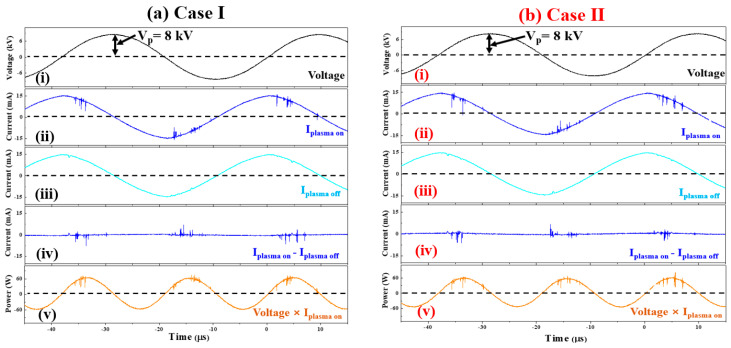
Characteristics of applied voltage (**i**), total current in plasma ON (**ii**) and OFF (**iii**) state, discharge current (**iv**), and instantaneous power (**v**) of the APPR with capillary electrodes measured under optimum conditions for Cases I (**a**) and II (**b**).

To monitor the electrical property of the discharge produced in the APPR with capillary electrodes for Cases I and II, the applied voltage and corresponding discharge currents were measured during discharge. Figure 5a,b show the applied voltage and total current measured when the discharge is sustained under optimal conditions for Cases I and II. As shown in the voltage waveform (ⅰ) in Cases I and II in Figure 5a,b, the AC sinusoidal voltage waveform applied to the capillary electrodes was not distorted by plasma discharge. The total current observed during the plasma-on state, shown in (ⅱ) of Cases I and II, consisted of the discharge and displacement currents. The discharge current was acquired by subtracting the displacement current obtained during the plasma-off state when the operating voltage was applied without discharge gas, an shown in (ⅲ) of Cases I and II. For all Cases I and II, the discharge current (I_Plasma ON_ − I_Plasma OFF_) was observed to be periodic, as shown in (ⅳ), implying that the discharge was regularly produced in a stable state. Moreover, the discharge current in the exposed electrode structure was observed to be distributed evenly in the positive and negative periods of the applied voltage when the instantaneous power delivery was at a high level above 45 W. 

In this case, the instantaneous power consumption is shown in (ⅴ) of Cases I and II, and the average power, P_a_, in APPRV is calculated from Equation (3).

(3) $$P_{a}=\frac{1}{T}\int_{0}^{T}V\left(t\right)\times I\left(t\right)dt,\;$$

where *T* is the applied voltage period, *V(t)* is the voltage signal, *I(t)* is the acquired current, and *t* is the time [37]. The average power during one period was calculated using the integrated value of the power waveform during one period. As a result, the average power values of the APPR with capillary electrodes in Cases I and II are similar, being around 2.45 W.

**Figure 6:**
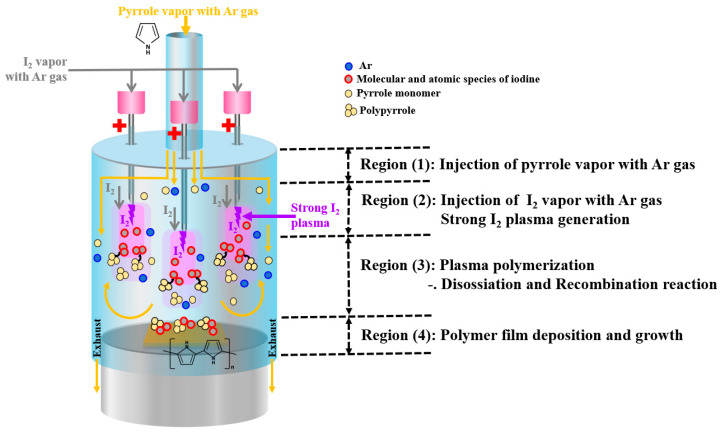
Schematic diagram of the proposed mechanism for in situ I_2_-doped PPy nanostructure film growth.

ICCD and OES measurement results revealed the mechanism of separately introducing a large amount of the heated I_2_ vapor flow from the monomer for the in situ I_2_-doped PPy nanostructure film growth (Figure 6). Plasma polymerization occurred inside the glass guide tube of the APPR. The glass guide tube was vertically divided into four regions (i.e., Regions (1)–(4)) to describe this plasma polymerization in the APPR (Figure 6). 

The processes that sequentially occurred in each region are as follows: first, the pyrrole vapor obtained from Ar gas bubbling was injected into the glass guide tube separately from the I_2_ vapor (Region (1)). Next, the heated I_2_ vapor went into the capillary electrodes together with the Ar gas, but separately from the pyrrole monomer. The injected I_2_ vapor generated an I_2_ plasma due to a high electric field induced by the nozzle electrodes, thus producing the reactive I_2_ species. The formed I_2_ species directly participated in in situ I_2_-doped PPy nanostructure film growth (Region (2)). In Region (3), the destruction of the pyrrole monomer was minimized due to the low-energy Ar plasma inside the glass guide tube, and the PPy polymer could be synthesized from the pyrrole monomer through various reactions, including monomer dissociation and recombination reactions. Subsequently, the I_2_ species could easily react with the PPy polymer during in situ I_2_-doped PPy nanostructure polymer film growth. Finally, the PPy polymer films in Region (4) were grown on the substrate by continuously repeating a series of plasma polymerizations occurring in Regions (1)–(4). The in situ I_2_-doped PPy nanostructure had a 15 µm thickness and good electrical conductivity. 

### 3.2. Characterization of the In Situ I_2_-Doped PPy Nanostructure Films 

#### 3.2.1. FTIR Spectra of the In Situ I_2_-Doped PPy Nanostructure Films

**Figure 7:**
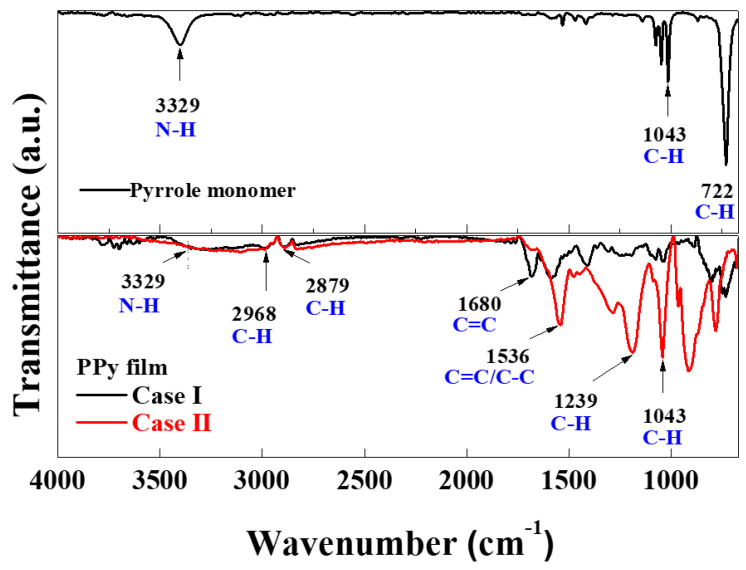
FTIR spectra of the pyrrole monomer and in situ I_2_-doped PPy nanostructure films grown in the APPR for Cases I and II under optimum conditions.

FTIR spectroscopy was employed to confirm the chemical structure of the in situ I_2_-doped PPy nanostructure films grown by APPR. Figure 7 presents the FTIR spectra of the pyrrole liquid monomer and in situ I_2_-doped PPy film nanostructure grown by the APPR with capillary electrodes on the silicon substrates for 30 min under optimum conditions for both Cases I and II. The peak at 3329 cm^−1^ in the FTIR spectrum of the pyrrole liquid monomer was assigned to the stretching vibration of the N–H bonds, while the peak at 1043 cm^−1^ originated from C–H in-plane band stretching. The peak at 722 cm^−1^ was assigned to the C–H out-of-plane stretching vibration in the pyrrole rings [47,48,49]. 

These peaks corresponding to the pyrrole monomers were also present in the FTIR spectra of the deposited PPy films. The characteristic peaks in the PPy film spectra were observed at 3329, 2968, 2879, 1680, 1536, 1239, and 1043 cm^−1^. The peak at 3329 cm^−1^ was assigned to N–H stretching [44,47]. The peaks at 2968 and 2879 cm^−1^, which were ascribed to aliphatic C–H stretching absorptions, indicated fragmentation of some pyrrole rings in the polymer [47,48,49]. The peaks at 1043, 1239, and 1536 cm^−1^ were assigned to C–H in-plane band stretching, C–N stretching, C=C, and C–C ring stretching, respectively [30,39,40,41,42,43,44,45,46,47,48]. 

Table 2 summarizes the detailed assignments of the absorption peaks in the FTIR spectra of the pyrrole monomer and in situ I_2_-doped PPy nanostructure films. The FTIR results show that the pyrrole ring was retained in Case II [44,47,48,49]. In these results, the peak intensities of the three major phases (1043, 1239, and 1536 cm^−1^) substantially enhanced compared to those in Case I owing to the increase in the excited N_2_ species through an ionization reaction between the N_2_ and electron species from the injected I_2_ vapor because of the I_2_ plasma [44,47,48,49]. The peak positions of the three major phases matched well with those reported previously for chemical based classical and plasma polymerized PPy [36,44,47,48,49]. In Case II, the peak intensity of the three phases of 1043, 1239, and 1536 cm^−1^ largely increased due to the increase in excited N_2_ species by I_2_ plasma through the ionization reaction between the N_2_ and electron species from the injected I_2_ vapor when compared to Case I [44]. Notably, the peak intensity increments of the three major phases were related to the improvement in the electrical conductivity of the PPy film through in situ I_2_ doping [36,44,47,48,49].

#### 3.2.2. Plane-View and Cross-Section FE-SEM Images of the PPy Nanostructure Films

**Figure 8:**
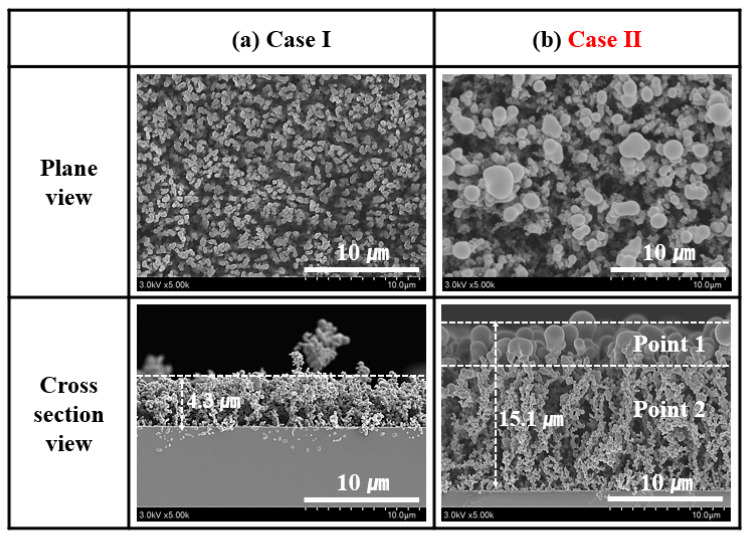
Plane-view and cross-section images of the PPy films grown on the silicon substrate for 30 min grown in the APPR under optimum conditions for the two cases, that is, (**a**) Cases I and (**b**) II.

Figure 8a,b present the plane-view and cross-section FE-SEM images of the PPy films grown on the silicon wafer for 30 min by the APPR at optimum conditions for the two cases of I_2_ doping. In Case 1 (no doping), small PPy nanoparticles (NPs) in spherical form were distributed on the PPy film surface (Figure 8a). In Case II (in situ I_2_ doping), the size of the spherical PPy NPs on the PPy film surface increased (Figure 8b). 

We obtained the cross-section of the FE-SEM images of the PPy film after its growth to determine its growth behavior and the change in its morphology owing to the heated I_2_ vapor. The film thicknesses in Cases I and II were approximately 4.3 and 15.1 µm, respectively (Figure 8a,b). The higher film thickness in Case II was presumably due to the increase in the N_2_ and I_2_ species because of the heated I_2_ vapor. In other words, this increase in the N_2_ and I_2_ species could be a critical factor affecting the film growth rate [45,46]. For Case II, the cross-section images showed that the in situ I_2_-doped PPy film with a cross-linked nanostructure grew in the vertical direction compared to those for Case I (Figure 8a,b). Compared to that in Case 1, the particle size in Case II was substantially different, with big particles being formed on the surface because of the heated I_2_ vapor (Figure 8a,b). This change in the surface and vertical structure of in situ I_2_-doped PPy nanostructure films contributes to the improvement in the film’s electrical conductivity properties [45,46]. 

**Figure 9:**
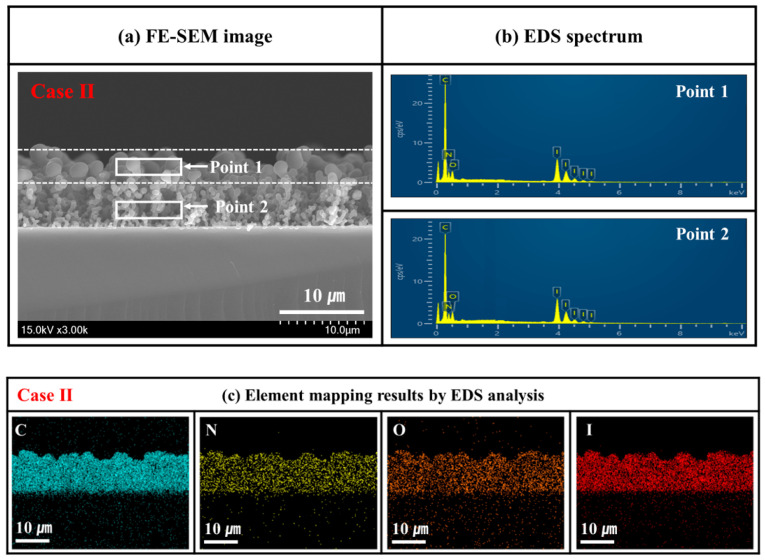
(**a**) FE-SEM images of the cross-section sample. (**b**) EDX spectra indicating the analysis positions. (**c**) Element mapping images obtained through EDX for the in situ I_2_-doped PPy nanostructure film (Case II) grown on a silicon wafer in the APPR for 30 min under optimum conditions.

To analyze the component distribution in the cross-section of the in situ heated I_2_-doped PPy nanostructure film (Case II), we measured its element distribution through EDX analysis. Figure 9a presents an FE-SEM cross-section image of the in situ heated I_2_-doped PPy nanostructure film (Case II). We measured the component spectra of the surface (Point 1) and inner (Point 2) regions based on the FE-SEM cross-section image (Figure 9a) and acquired the element mapping images of C, N, O, and I elements (Figure 9b,c). Figure 9b presents the EDX spectra of the two analysis regions. Table 3 presents the element compositions obtained based on these EDX spectra for two different regions. It was confirmed that the component composition for each region was similar, that is, no difference was found between the element distributions of the surface and inner regions in the in situ I_2_-doped PPy nanostructure film (Case II). Figure 9c shows the element mapping images of C, N, O, and I elements in the cross-section of the in situ I_2_-doped PPy nanostructure film (Case II) grown by the APPR. Figure 9b,c and Table 3 confirm that C, N, O, and I were uniformly distributed in the cross-section of the in situ I_2_-doped PPy nanostructure films (Case II). The FE-SEM and EDS results showed no difference between the components of the surface and inner regions. Only the surface particle size of the in situ I_2_-doped PPy nanostructure film changed owing to the temperature effect during the injection of the heated I_2_ vapor. The formed I_2_ species could directly participate in in situ I_2_-doped PPy film growth, thereby allowing them to be homogeneously distributed in the PPy films [14].

#### 3.2.3. Electrical Conductivity of the In Situ I_2_-Doped Ppy Nanostructure Film

The electrical conductivities (σ) of the Ppy nanostructure films grown on glass substrates by the APPR for both Cases I and II. For Case I, the electrical σ could not be measured owing to a high electrical R_s_ over a few tens MΩ/sq, which was out of range for instrument measurement. Meanwhile, the electrical conductivities (σ) of the PPy nanostructure films grown at different heating conditions (room temperature and 35 °C) were measured to be 3.36 × 10^−4^ S/cm and 5.86 × 10^−4^ S/cm, respectively. Table 4 shows the changes in I_2_ weight used for the in situ doping I_2_ process relative to heating temperature. The change in the I_2_ weight means those in the I_2_ vapor used for the doping process, even if the changes in the weight of I_2_ before and after are not exactly identical to those in the I_2_ vapor used for the experiment. As I_2_ vapor increases approximately twice, the corresponding conductivity of the PPy nanostructure film increases by about 74%. However, at 50 °C, the grown film was observed to be a condensed phase film (not shown here). In addition to an increase in the I_2_ vapor, the thickness, connection of cross-linked PPy polymer NPs, and change in surface morphology also contribute to obtaining the higher conductivity in Case II [45,46].

**Figure 10:**
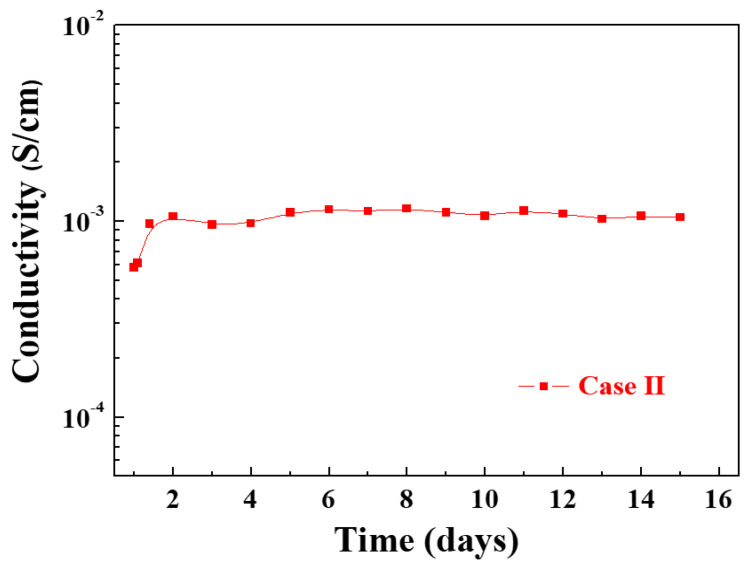
Measured changes in the electrical σ of in situ I_2_-doped PPy nanostructure film (Case II) for 14 days.

We investigated the electrical σ of the in situ I_2_-doped PPy nanostructure film (Case II) grown by the APPR as a function of exposure time (days) at room temperature and ambient air conditions with a humidity of approximately 40% to check its long-term stability. Figure 10 shows the changes in the electrical σ over time for the in situ I_2_-doped PPy nanostructure film (Case II) on glass substrates grown by the APPR. The electrical σ of the in situ I_2_-doped PPy film (Case II) was initially increased from 5.83 × 10^−4^ to 9.69 × 10^−4^ S/cm after a 1 day air exposure. After that, the electrical σ value tended to be well maintained in the 1.0 × 10^−3^ S/cm range for 14 days under ambient conditions after more than 5 days of air exposure. The reason would be considered that during the 1 day, the electrical σ of the in situ I_2_-doped PPy nanostructure film (Case II) initially increased owing to the carriers doping by adsorption of oxygen or H_2_O in the ambient air [50,51]. When exposed to air for a long time, the electrical σ does not continuously increase and is maintained because the presence of H_2_O in the surrounding atmosphere passivates the dopants in the polymer film [51].

## 4. Conclusions

This work investigated the in situ I_2_-doped PPy nanostructure film grown in an APPR with capillary electrodes, wherein a large amount of heated I_2_ vapor was separately injected from the monomer during the polymer film growth. For in situ I_2_-doped PPy nanostructure film growth, the bluff body position was optimized with respect to the guide tube in the APPR. Based on experimental results, we obtained the optimum bluff body position of 15 mm, which produced intense plasma for the in situ heated I_2_-doped PPy film growth under Ar flow rates of 1000 sccm and 300 sccm for the heated I_2_ and pyrrole vapors, respectively. OES and ICCD analyses confirmed that the I_2_ vapor injected through the capillary nozzle electrode forms an I_2_ charge species near the capillary nozzle electrode based on the I_2_ plasma produced by a high electric field induced by the nozzle electrode. The formed I_2_ species could directly participate in growing in situ I_2_-doped PPy nanostructure films. For the two cases, that is, no doping (Case I) and in situ I_2_-doped PPy nanostructure film (Case II), we investigated the characteristics of the PPy film grown in the APPR under optimum conditions. FE-SEM results confirmed that the in situ I_2_-doped PPy nanostructure films (Case II) uniformly grew in a cross-linked nanostructure in the vertical direction with NPs and 15.1 μm thickness. Only the surface particle size of the in situ heated I_2_-doped PPy nanostructure film changed owing to the injection of the heated I_2_ vapor. The formed I_2_ species could directly participate and easily react with the PPy during in situ I_2_-doped PPy nanostructure film growth, allowing them to be homogeneously distributed in the PPy films. Thus, the in situ I_2_-doped PPy nanostructure film (Case II) showed a good electrical σ value of 5.83 × 10^−4^ S/cm and a long-term conductivity stability feature compared to the corresponding non-doped film (Case I). 

## Figures and Tables

**Table 1:** Detailed experimental conditions for synthesizing in situ I_2_-doped PPy nanostructure films using the proposed APPR.

Electrode type	Three capillary electrodes
Precursor material	Pyrrole liquid monomer
Driving voltage waveform	AC sinusoidal
Driving voltage (V_p_)	8 kV
Frequency	26 kHz
Ar gas flow for pyrrole vapor	300 sccm
Ar gas flow for heated iodine vapor	1000 sccm
Hot plate temperature for I_2_ vapor	35 °C
Deposition time	30 min
Bluff body height	H = 10, 15, and 20 mm (controllable)
Distance between electrode tip and substrate	D = 45, 40, and 35 mm (controllable)
Two cases of in situ I_2_ doping	Case I: no-doping
	Case II: in situ I_2_ doping at 35 °C

**Table 2:** FTIR peak assignments for the pyrrole monomer and in situ I_2_-doped PPy nanostructure films grown in the APPR on the silicon wafer under optimum conditions.

	Wave Number	Peak Assignment
	722 cm^−1^	C–H out of plane stretching
Pyrrole monomer	1043 cm^−1^	C–H in-plane bend stretching
	3329 cm^−1^	N–H stretching
	1043 cm^−1^	C–H in-plane-bend stretching
	1239 cm^−1^	C–N stretching
	1536 cm^−1^	C=C, C–C ring stretching
PPy film	1680 cm^−1^	C=C stretching
	2879 cm^−1^	C–H asymmetric stretching
	2968 cm^−1^	C–H symmetric stretching
	3329 cm^−1^	N–H stretching

**Table 3:** Elemental composition obtained through EDX for the in situ I_2_-doped PPy nanostructure film (Case II) grown on the silicon substrates in the APPR.

	Elemental Composition (Atomic %)				
	C	N	O	I	Total
Point 1 (top, big particle)	69.9	20.1	4.8	5.2	100
Point 2 (inner, small particle)	69.0	19.6	5.7	5.7	100

**Table 4:** Changes in I_2_ weight used for the in situ I_2_ doping process relative to heating temperature.

Heating Temperature (°C)	I_2_ Weight (g)		I_2_ Weight (g) Used for Process
	Before	After 30 min	
Room temperature	10	9.9	0.1
35	10	9.8	0.2
50	10	9.7	0.3

## Data Availability

Data are contained within the article.
